# Significant Short-Term Shifts in the Microbiomes of Smokers With Periodontitis After Periodontal Therapy With Amoxicillin & Metronidazole as Revealed by 16S rDNA Amplicon Next Generation Sequencing

**DOI:** 10.3389/fcimb.2020.00167

**Published:** 2020-05-05

**Authors:** Daniel Hagenfeld, Johannes Matern, Karola Prior, Inga Harks, Peter Eickholz, Katrin Lorenz, Ti-Sun Kim, Thomas Kocher, Jörg Meyle, Doğan Kaner, Ulrich Schlagenhauf, Dag Harmsen, Benjamin Ehmke

**Affiliations:** ^1^Department of Periodontology and Operative Dentistry, Münster University Hospital, Münster, Germany; ^2^Department of Periodontology, Johann Wolfgang Goethe-University Frankfurt, Frankfurt, Germany; ^3^Department of Periodontology, TU Dresden, Dresden, Germany; ^4^Section of Periodontology, Department of Conservative Dentistry, University Hospital Heidelberg, Heidelberg, Germany; ^5^Unit of Periodontology, University Medicine Greifswald, Greifswald, Germany; ^6^Department of Periodontology, University of Giessen, Giessen, Germany; ^7^Department of Periodontology, Dental School, Faculty of Health, University of Witten/Herdecke, Witten, Germany; ^8^Departments of Periodontology and Synoptic Dentistry, Charité University Medicine Berlin, Berlin, Germany; ^9^Department of Periodontology, University Hospital Würzburg, Würzburg, Germany

**Keywords:** microbiota, smoking, periodontal therapy, systemic antibiotics, 16S rDNA amplicon sequencing

## Abstract

The aim of this follow-up study was, to compare the effects of mechanical periodontal therapy with or without adjunctive amoxicillin and metronidazole on the subgingival microbiome of smokers with periodontitis using 16S rDNA amplicon next generation sequencing. Fifty-four periodontitis patients that smoke received either non-surgical periodontal therapy with adjunctive amoxicillin and metronidazole (n = 27) or with placebos (n = 27). Subgingival plaque samples were taken before and two months after therapy. Bacterial genomic DNA was isolated and the V4 hypervariable region of the bacterial 16S rRNA genes was amplified. Up to 96 libraries were normalized and pooled for Illumina MiSeq paired-end sequencing with almost fully overlapping 250 base pairs reads. Exact ribosomal sequence variants (RSVs) were inferred with DADA2. Microbial diversity and changes on the genus and RSV level were analyzed with non-parametric tests and a negative binomial regression model, respectively. Before therapy, the demographic, clinical, and microbial parameters were not significantly different between the placebo and antibiotic groups. Two months after the therapy, clinical parameters improved and there was a significantly increased dissimilarity of microbiomes between the two groups. In the antibiotic group, there was a significant reduction of genera classified as *Porphyromonas, Tannerella*, and *Treponema*, and 22 other genera also decreased significantly, while *Selenomonas, Capnocytophaga, Actinomycetes*, and five other genera significantly increased. In the placebo group, however, there was not a significant decrease in periodontal pathogens after therapy and only five other genera decreased, while *Veillonella* and nine other genera increased. We conclude that in periodontitis patients who smoke, microbial shifts occurred two months after periodontal therapy with either antibiotics or placebo, but genera including periodontal pathogens decreased significantly only with adjunctive antibiotics.

## Introduction

Periodontitis is a highly prevalent, biofilm-associated disease and the main reason for tooth loss in the elderly population (Frencken et al., 2017). In a large multi-center study (ABPARO) a statistically significant benefit of amoxicillin (500 mg) and metronidazole (400 mg) as an adjunct to periodontal therapy was investigated and clinical results were published (Harks et al., 2015). We recently performed a microbial sub-group analysis of non-smoking periodontitis patients from the ABPARO study (Hagenfeld et al., 2018). We showed that the used antibiotics caused a microbiome shift characterized by the reduction of the amounts of periodontal pathogens and increase of commensal bacteria in the subgingival biofilm, 2 months after periodontal therapy. Cigarette use is a major risk factor for destructive forms of periodontal disease and profoundly influences the subgingival microbiome, which means that it is more diverse, pathogen rich, and commensal poor (Winkelhoff et al., 2001; Shchipkova et al., 2010; Bizzarro et al., 2013; Moon et al., 2015), even in shallow periodontal pockets ≤4 mm (Haffajee and Socransky, 2001). Re-colonization with commensals after periodontal therapy is compromised in smokers, because they maintain a pro-inflammatory host phenotype (Joshi et al., 2014).

Metronidazole interferes with the nucleic acid synthesis of bacteria. It has an antimicrobial spectrum against anaerobic bacteria including the periodontal pathogens *Porphyromonas ginigvalis, Tannerella forsythia*, and *Treponema denticola* (Loesche et al., 1984). Amoxicillin inhibits the synthesis of bacterial cell walls and has a synergistic effect together with metronidazole on the reduction of *Aggregatibacter actinomycetemcomitans* that is associated with aggressive forms of periodontitis (van Winkelhoff et al., 1989). Currently, there are only a few studies that have examined the microbial community effects of adjunctive amoxicillin and metronidazole in the periodontal treatment of smokers (Matarazzo et al., 2008; Faveri et al., 2014). Those studies, however, detected only a pre-defined and limited number of bacteria with the DNA-DNA checkerboard method. So-called “next-generation sequencing” allows for a hypothesis-free and unrestricted view on the microbial dynamics, because no pre-selection of bacteria is needed.

To date, there has been no next generation sequencing study that has investigated the microbial dynamics of smokers after therapy with or without antibiotics. Therefore, the aim of this study was to compare the effects of mechanical periodontal therapy with or without adjunctive amoxicillin and metronidazole on the subgingival microbiome of patients that smoke and have periodontitis using 16S rDNA amplicon next generation sequencing.

## Materials and Methods

### Patient Characteristics

Specimens from the ABPARO study—a multicenter randomized, double-blinded, parallel group, placebo-controlled study on the effects of adjunctive antibiotics during periodontal treatment—were used (ISRCTN: 64254080, Clinical Trials.gov NCT00707369) (Harks et al., 2015). Smokers were defined as persons self-declared as smokers and having a concentration of carbon-monoxide (CO) ≥ 7 ppm by using a Smokerlyzer (Bedfont-Smokerlyzer®, Bedfont, UK) (Harks et al., 2015). All smokers received the same mechanical periodontal treatment. In total, 71 of the 345 per-protocol patients were smokers. Of those, nine were diagnosed with severe (Stage III) and 62 with moderate periodontitis (Stage II) (Tonetti et al., 2018). Seven severely and 27 moderately diseased smokers received an adjunctive placebo, and two severely and 35 moderately diseased smokers received adjunctive amoxicillin and metronidazole (500 mg/400 mg thrice daily for 7 days). Because of the small and unbalanced treatment groups of severely diseased smokers, only the data from the smokers with moderate periodontitis were included in this study and reported here. To ensure the best clinically comparable groups at baseline, the 27 smokers with moderate periodontitis from the placebo group were matched to 27 of the 35 moderately diseased smokers from the antibiotic group (Ho et al., 2011). Samples of those 54 smokers were analyzed for the examination time-points before and 2 months after therapy. Subgingival specimens were taken from four teeth with periodontitis and a pocket probing depth of ≥6 mm, one in each quadrant, as described previously (Hagenfeld et al., 2018, 2019). One sterile paper point (ISO45, Roeko Dental, Langenau, Germany) was inserted for 10 s in each site and all paper points were removed and pooled in one sterile collection tube. The sampling sites remained the same for both sampling timepoints. This study was approved by the Medical Ethics Committee of the University of Muenster (ref: 2016-505-f-S).

### Library Preparation

Bacterial genomic DNA from the samples of smokers and non-smokers was isolated and purified using the QiaAmp DNA- Mini Kit (Qiagen, Hilden, Germany). The integrity and purity of the DNA was checked by agarose gel electrophoresis and a Nanodrop (Thermo Scientific, Silverside, USA) measurement, respectively. DNA quantification was performed with a Qubit 2.0 fluorometer together with the Qubit dsDNA BR Assay Kit (Thermo Fisher, Waltham, MA, USA). Two PCRs were done with KAPA HiFi Hot Start DNA Polymerase (Ready Mix, KAPA-Biosystems, Boston, MA, USA). In the first PCR, universal eubacterial tailed tag dual index amplification primers with an amplicon size of 291 bp (*E. coli*-position 515 to 806) were utilized for amplifying the V4 hypervariable regions of the bacterial 16S rRNA gene (Klindworth et al., 2013): EMP515f TCGTCGGCAGCGTCAGATGTGTATAAGAGACAG**GTGYCAGCMGCCGCGGTAA**; 806_R GTCTCGTGGGCTCGGAGATGTGTATAAGAGACAGGACTACHV**GGGTATCTAATCC**. The 16S rDNA specific regions were marked as bold. In the second PCR, sample-specific “barcode”-primers and adapter sequences were attached using the Nextera XT Index Kit Version 2 (Illumina, San Diego, CA, USA) with 96 barcode combinations. Up to 96 libraries were normalized and pooled for an Illumina MiSeq sequencing run with 250 base-pair paired-end reads using MiSeq Reagent Kit Version 2 (Illumina). Five negative control samples contained molecular biology grade water which allowed for the identification of potential external contamination in each sequencing run. Additionally, one evenly balanced mock sample supplemented each run with 23 known species with 24 RSVs to allow for inter-run quality control. The composition of the mock sample can be found in Supplementary File 1. The DNA extraction steps for the mock sample were performed as described earlier with one modification: the DNA isolation for the mock species was performed with Qiagen Genomic Tips 20/G (Qiagen, Hilden, Germany). All 23 single-species samples were adjusted to 10^7^ copies of the 16S rRNA operon per organism per microliter. Afterwards, an even mock sample was created by combining equal amounts of these dilutions with a total copy number of 10^7^ 16S rRNA operons per microliter resulting in 4.3 × 10^5^ 16S rRNA operons per species per microliter.

### Sequence Generation, Primer, and Adapter Removal

lllumina's MiSeq Control Software v.2.6.2.1 was used to operate the MiSeq machine. The Real-time Analysis Software v.1.18.54 performed image analysis, base calling, and quality score assignment to each base for each cycle. The MiSeq Reporter Software v.2.6.3 was used for demultiplexing, FastQ file generation, and adapter removal. Sequencing primers were removed with Cutadapt v.1.8.1 (Martin, 2011) using the default setting, that is, a maximum error rate in the adapter region of 10%. Those trimmed reads were submitted to the European Nucleotide Archive (http://www.ebi.ac.uk/ena/) of EMBL European Bioinformatics Institute under the study accession number PRJEB35812.

### Denoising and Merging

The adapter and primer-free FastQ files were further processed using R v.3.6.1 and RStudio v.1.1.463 (R Development Core Team, 2017) with DADA2 v.1.12.1 (Callahan et al., 2016). If not mentioned explicitly below, the default settings (most noticeably, a zero-mismatch allowed in the overlap region while merging the forward and reverse reads) were used. First, the forward and reverse reads were trimmed one base at the 3′-end to remove the terminal base without the quality score that was attached during sequencing. Second, the reads were trimmed at the 5′-end to allow for merging with minimal unpaired bases at both ends calculated for *E. coli* (forward reads were trimmed 22 bases, reverse reads 20 bases). Third, a maximum of two expected errors per read was allowed to filter out low-quality reads. Finally, the minimal abundance was set to 10 reads per run for forming the ribosomal sequence variant (RSV) partitions during denoising.

### Taxonomic Labeling and Decontamination

Taxonomic labeling of RSVs was done with a naïve Bayesian classifier and a SILVA v.128 training set (Pruesse et al., 2007). Each run was stored using phyloseq v.1.19.1 (McMurdie and Holmes, 2013) into a single file containing taxonomic labels and reads for each RSV. The QC-checked sequencing runs were individually screened for potential external contaminants, using the decontam package v.1.4.0 (Davis et al., 2018). The combined prevalence and frequency methods were used with the default threshold of 0.1 for identifying contaminants. Afterwards, all single-run phyloseq files were merged and combined with clinical and demographic variables into the final phyloseq file. Finally, prevalence filtering over all the runs was done of RSVs occurring in only two or less samples to remove spurious ones.

### Run and Sample Quality Control

Run quality control was performed after each sequencing run in two consecutive steps. First, the run-Q30 scores were checked with the MiSeq Reporter software. Here, the values had to be above 75% as recommended by the manufacturer (Illumina, 2012). Second, the mock sample was denoised using the DADA2 pipeline and checked with phyloseq. A run was judged of sufficient quality when 24 found RSVs completely matched the reference 16S RNA genes of each 23 species from the mock sample and ≤ 0.5% reads per sample were found without matching references. Sample quality control was first performed for all samples after each run and again a second time after the final phyloseq file with all included runs was created. Samples had insufficient quality when the rarefaction quality control did not show saturation or the samples had ≤5,000 reads.

### Statistical Analysis

For the community level analysis, phyloseq was used to analyze the bacterial diversity. For alpha diversity, the richness, that is, the randomly sub-sampled number of observed reads per RSVs in each sample, was determined. In addition, the Shannon index for diversity was provided. For beta diversity, a Bray-Curtis distance matrix was created, and the mean dissimilarity for each patient was calculated. Fisher's exact test was used for the intergroup differences of the categorical variables. For all other continuous variables, the two-sided Mann–Whitney *U*-test for intergroup differences was used. For the changes of the continuous variables in both groups before and after the treatment, the Wilcoxon signed-rank test was applied.

The differences in the mean number of randomly subsampled RSV reads per genus between the antibiotic and placebo before and after therapy were displayed in a heatmap. For the statistical analysis of the differential abundance of single RSVs classified on genus level, the R package DESeq2 v.1.18.1 (Love et al., 2014) was used. The model formula contained the treatment-group (AB, placebo), the timepoint (before, after), and the interaction term between both groups as fixed effects. The RSVs grouped per genus that changed significantly in the antibiotic and placebo groups were displayed in a bubble chart. If one genus included species associated with periodontal disease or health by the Socransky group (Socransky et al., 1998), this genus was allocated to the given complex as described earlier (Hagenfeld et al., 2018).

## Results

### Demographic, Clinical Parameters Before and After Therapy and Preprocessing the Sequencing Output

Before therapy, there were no statistically significant differences between the placebo and antibiotic groups in all the demographic and clinical parameters examined (Table 1). After therapy, clinical parameters improved significantly in both groups (Supplementary Table 1, Supplementary File 2). All sequencing runs passed the run quality control as described previously (Supplementary Table **3**). A total of five RSVs were found to be suspicious for external contaminants and removed. In addition, 645 RSVs had a prevalence in ≤2 samples and were also removed, together with two RSVs not classified as from the bacterial kingdom. All samples showed a saturation during rarefaction and had more than 5,000 reads per sample (data not shown).

**Table 1:** Clinical and demographic variables for the placebo and antibiotic groups before therapy.

		**Placebo** **(*n* = 27)**	**Antibiotic** **(*n* = 27)**	***p*-value**
Sex	Female	15	16	*p* = 1.000
	Male	12	11	
	<45	11	8	*p* = 0.5131
Age	45 ≤ 55	13	13	
	> 55	3	6	
	Mean and sd	24.48 ± 13.13	21.67 ± 13.38	*p* = 0.3632
%PD 5 mm	Median	25.00	19.00	
	1st, 3rd Quantile	13.00, 35.00	13.00, 26.00	
	Mean and sd	31.70 ± 14.95	28.04 ± 14.64	*p* = 0.3677
%Bleeding	Median	30.00	30.00	
	1st, 3rd Quantile	22.50, 39.50	21.50, 35.00	

*Sex, age, percentage of pocket depths ≥ 5 mm, and percentage of sites with bleeding for the placebo and antibiotic groups. Categorical values were tested for uneven distribution at baseline with Fisher's exact test; for continuous variables, the Mann–Whitney U-test was used*.

### Microbial Diversity Parameters Before and After Therapy

Before therapy, there were no statistically significant differences between the placebo and antibiotic groups in any of the microbial diversity parameters tested: mean richness (placebo 161.81 ± 53.27 sd vs. antibiotic 169.93 ± 57.32 sd, *p* = 0.595), mean diversity (placebo 3.84 ± 0.39 vs. antibiotic 3.86 ± 0.41, *p* = 0.797), and mean dissimilarity (placebo 3.84 ± 0.39 vs. antibiotic 3.86 ± 0.41, *p* = 0.757). After therapy, the dissimilarity increased significantly in both the placebo (*p* = 0.034) and the antibiotic group (*p* < 0.001). Richness (*p* = 0.768) and diversity (*p* = 0.090) were not significantly altered in the placebo group. In the antibiotic group, however, richness (*p* = 0.002) and diversity (*p* = 0.016) significantly decreased. A detailed table of diversity parameters can be found in Supplementary Table 2.

### Genus Level Abundances, Before, and After Therapy

**Figure 1:**
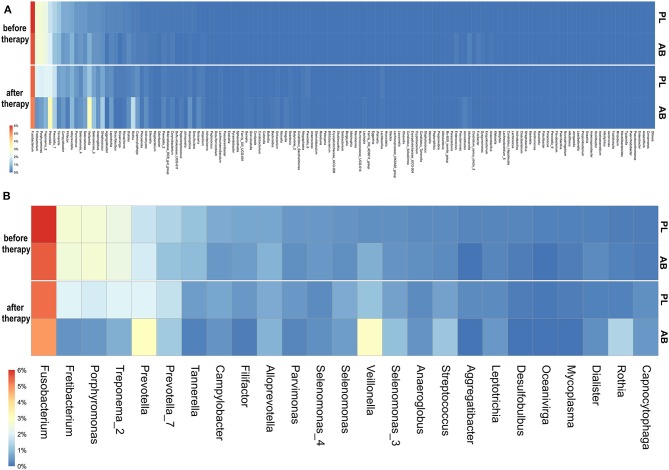
**(A)** Heatmap showing the randomly sub-sampled reads summarized per genus in the placebo and antibiotic groups before and after therapy. The color codes ranged from 6% to 0% of the summarized reads for each genus. Higher relative numbers of genus reads are colored red, lower numbers are yellow to white, and dark-blue represents zero values. PL, placebo group; AB, antibiotic group. **(B)** Heatmap showing randomly sub-sampled reads of the 25 most abundant genera in the placebo and antibiotic groups before and after therapy. Here, only the 25 most abundant genera before therapy are shown. The color codes ranged from 6 to 0% of the summarized reads for each genus. Higher relative numbers of genus reads are colored red, lower numbers are yellow to white, and dark-blue represents zero values. PL, placebo group; AB, antibiotic group.

We found in total 1060 RSVs that can be assigned to 143 different genera (Supplementary File 3). Before therapy, the relative number of sub-sampled reads summarized by per genus was highly similar between the placebo and the antibiotic groups (Figure 1).

Before therapy, no genus was more dissimilar than 1.0 percent between antibiotic and placebo. Those RSVs with the highest differences before therapy were *Fusobacterium, Veillonella*, and *Prevotella*, all below a 0.5 percent difference. The three genera with the highest relative abundance before therapy were: *Fusobacterium* (placebo: 6.0% vs. antibiotic: 5.5%), *Veillonella* (placebo: 0.4% vs. antibiotic: 0.8%), and *Prevotella* (placebo: 1.4% vs. antibiotic: 1.1%).

After therapy, the microbiomes became more dissimilar between the placebo and antibiotic groups. The genus with the highest difference after therapy was *Veillonella*, which increased after therapy in both groups and had a relative abundance of 1.1% in the placebo group, compared to 1.9% in the antibiotic group. *Fretibacterium* and *Porphyromonas* decreased less in the placebo group; they had a relative abundance of 2.0 and 1.9% in the placebo vs. 0.4 and 0.5% in the antibiotic group, respectively. In the placebo group, no genus changed more strongly than one percent of relative abundance. In the antibiotic group, six genera changed more than one percent. Of those, three genera (*Fretibacterium, Porphyromonas*, and *Treponema*) reduced and three genera increased more than 1.0% (*Veillonella, Rothia*, and *Prevotella*). In the placebo group, also mainly periodontal pathogens were reduced and commensals increased after therapy. However, those changes were in general roughly 0.5–1.5% smaller than in the antibiotic group.

### RSVs Grouped at Genus Level That Significantly Changed After Therapy

**Figure 2:**
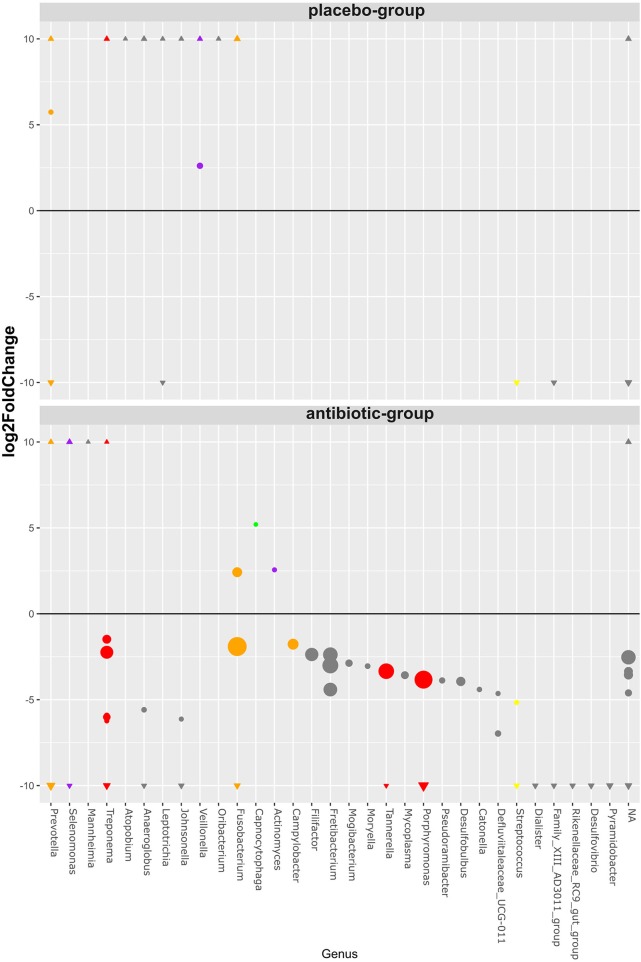
Bubble chart of significant RSV abundance changes after periodontal therapy classified on the genus level. The bubbles represent 98 RSVs, belonging to 31 uniquely named genera (*x*-axis), that showed statistically significant changes (*y*-axis) after therapy on a log2 scale. All RSVs unclassified on the genus level are grouped together (NA). The sizes of the bubbles represent the mean relative RSV abundance over all samples before therapy. Those RSVs with ≥10 log2fold and ≤ −10 log2fold changes were marked as triangles on their respective *y*-axis section. All RSVs belonging to a genus that includes species previously described by Socransky are colored according to their complex affiliation.

The vast majority (98.8%) of all RSVs were not statistically different in abundance between the antibiotic and placebo group before therapy. Those 21 RSVs that were found different in abundance before therapy belonged to exclusively low-abundant RSVs with a mean baseline abundance below 1% of each sample (Supplementary File 4). The abundances of RSVs from genera including periodontal pathogens like *Porphyromonas, Tannerella*, and *Treponema* decreased significantly only in the antibiotic group, together with several other species (Figure 2). RSVs belonging to genera from the commensal bacteria *Selenomonas, Capnocytophaga*, and *Actinomyces* significantly increased after therapy only in the antibiotic group. Two RSVs belonging to the genus *Veillonella* increased only in the placebo group, together with several other RSVs not belonging to formerly identified commensal species.

## Discussion

By using our hypothesis-free next-generation-sequencing approach, we have provided the first study that examined in-depth microbial changes in smokers after periodontal therapy with or without antibiotics. We found a short-term microbiome shift in the form of a significantly increased beta-diversity after therapy in both the placebo and antibiotic group. Those microbial shifts differed markedly between the placebo and antibiotic group. In the antibiotic group, the microbial shift was characterized by a strong reduction of alpha-diversity in general, and especially, periodontal pathogens decreased significantly while commensals increased. In the placebo group, however, there was not a significant decrease in alpha-diversity and also not a significant reduction of periodontal pathogens 2 months after therapy.

Compared to our previous study with non-smokers (Hagenfeld et al., 2018), the composition of highly abundant genera at baseline had a strong resemblance with the smokers in this study. In both studies, *Fusobacterium, Fretibacterium*, and *Porphyromonas* had the highest baseline abundance. Regarding RSV changes after therapy, we also had very similar results on the genus level compared to our study conducted with non-smokers; RSVs belonging to genera containing periodontal pathogens like *Treponema, Porphyromonas, Tannerella*, and *Fusobacterium* decreased as in our previous study with non-smokers. Additionally, in both studies, *Fretibacterium* and *Fillifactor* decreased among several other RSVs classified as genera not regarded as periodontal pathogens by the fundamental study of Socransky et al. (1998). Also, in both studies, RSVs that were classified as genera containing commensal species (*Selenomonas, Capnocytophaga*, and *Actinomyces*) increased in the antibiotic group. In the placebo group of non-smokers, however, we did not find dissimilarity changes, and secondly, only three RSVs changed significantly in the placebo group of our previous study with non-smokers.

One previous study (Matarazzo et al., 2008) that used the DNA-DNA checkerboard method is very comparable to our study, because they made a direct comparison of two groups of periodontitis patients who smoked that received either periodontal therapy with two different kinds of antibiotics or periodontal therapy without antibiotics. In that study, patients from the amoxicillin and metronidazole group underwent similar microbial shifts as in our study. Here, also periodontal pathogenic species with the genera *Porphyromonas, Tannerella*, and *Treponema* were significantly reduced only in the antibiotic group, accompanied by an increase of the species from commensals, that are, *Actinomyces, Selenomonas*, and *Capnocytophaga*.

Another study using the DNA-DNA checkerboard method compared the use of amoxicillin and metronidazole between smokers and non-smokers. Here, the smokers treated with adjunctive antibiotics also had a significant reduction of those three genera containing periodontal pathogens (Faveri et al., 2014).

Other groups that examined periodontal therapy without antibiotics in smokers and non-smokers found a lower reduction of periodontal pathogens in smokers compared to non-smokers by using the DNA-DNA checkerboard (Darby et al., 2005; Feres et al., 2015). These findings were confirmed by our results from the smokers in the placebo group where the relative numbers of high-abundant bacteria in general were slightly reduced, but no RSVs from periodontal pathogens were significantly changed after therapy.

In contrast to those previous hypothesis-driven studies, the majority of genera (55%) that were reduced significantly in our study with patients that smoked and received adjunctive antibiotics were not regarded as periodontal pathogens (Socransky et al., 1998). For example, uncultivated *Fretibacterium* sp. *human oral taxon no. 360* was recently found as a novel biomarker for periodontitis in a Japanese population (Khemwong et al., 2019). RSVs from the genus *Fretibacterium* were also highly abundant in our population of German smokers and decreased significantly after periodontal therapy with antibiotics. Another example is *Filifactor alocis* that is regarded as a new emerging periodontal pathogen (Aruni et al., 2014), or *Mogibacterium* as a new genus isolated from periodontal pockets of adult human patients with periodontal disease and infected root canals (Nakazawa et al., 2000). Both of those genera were reduced significantly in our study using antibiotics. These findings point out the importance of those and other previously unidentified species in the treatment of periodontal disease, as recently reviewed (Perez-Chaparro et al., 2014).

Our study has also some limitations. A higher taxonomic resolution down to the species level was not possible with our 250-bp read length. We could not access functional capacities of the microbiomes as would be possible with a meta-genomic approach. In addition, the negative binomial regression model used for the RSV-level changes cannot account for random patient effects. By using a pooled microbial sample, we cannot asses site specific differences. We only included moderately diseased periodontitis patients. A longer follow-up would also be needed to assess the stability of changes and patterns of re-colonization after therapy.

## Conclusions

With our hypothesis-free 16S rDNA amplicon next generation sequencing study with periodontitis patients that smoke, we found RSVs from genera including periodontal pathogens exclusively decreased with adjunctive amoxicillin and metronidazole. Additionally, we expanded the spectrum of bacteria that changed in short-term after periodontal therapy in smokers beyond the well-studied periodontal pathogens and commensals.

## Data Availability Statement

The datasets generated for this study can be found in the European Nucleotide Archive (http://www.ebi.ac.uk/ena/) of EMBL European Bioinformatics Institute PRJEB35812.

## Ethics Statement

The studies involving human participants were reviewed and approved by Medical Ethics Committee of the University of Muenster (ref: 2016-505-f-S). The patients/participants provided their written informed consent to participate in this study.

## Author Contributions

DHag and JMa contributed to formal analysis, validation, software, and methodology. DHag and KP contributed to investigation and data curation. BE and DHar contributed to project administration, conceptualization, and supervision. BE, IH, and DHar contributed to funding acquisition. PE, KL, T-SK, TK, JMe, DK, US, and BE contributed to resources. DHag contributed to Writing - original preparation. JMa, JMe, KP, IH, PE, KL, T-SK, TK, DK, US, BE, and DHar contributed to Writing - Review and Editing.

## Conflict of Interest

The authors declare that the research was conducted in the absence of any commercial or financial relationships that could be construed as a potential conflict of interest.
