# Supplementary material for: Significant Short-Term Shifts in the Microbiomes of Smokers With Periodontitis After Periodontal Therapy With Amoxicillin & Metronidazole as Revealed by 16S rDNA Amplicon Next Generation Sequencing
Source: Front Cell Infect Microbiol. 2020 May 5;10:167. doi: 10.3389/fcimb.2020.00167 (PMC7232543; doi:10.3389/fcimb.2020.00167)
Supplement: Supplementary file 1 [file Data_Sheet_1.docx]

**SUPPLEMENTARY TABLES:**

**Supplementary Table 1.:** Clinical variables for the placebo and antibiotic group before and after therapy

|  | **Placebo** n=27 | | | **Antibiotic** n=27 | | |
| --- | --- | --- | --- | --- | --- | --- |
|  | **Baseline** | **2 months after therapy** | ***p*-values** | **Baseline** | **2 months after therapy** | ***p*-values** |
| **%PPD5mm** | 24.48 ± 13.13 | 15.74 ± 11.22 | ***p*<0.001** | 21.67 ± 13.38 | 5.81 ± 5.78 | ***p*<0.001** |
|  | 25.00 (13.00 /  35.00) | 13.00 (8.00 / 20.50) |  | 19.00 (13.00/26.00) | 4.00 (1.50/8.50) |  |
| **%Bleeding** | 31.70 ± 14.95 | 21.41± 16.39 | ***p*=0.004** | 28.04 ± 14.64 | 8.00 ± 7.32 | ***p*<0.001** |
|  | 30.00 (22.50 / 39.50) | 17.00(11.50 / 25.50) |  | 30.00 (21.50 / 35.00) | 6.00 (2.00 / 11.00) |  |

Percentage of pocket depths ≥5mm and percentage of sites with bleeding, for the placebo and antibiotic group. Presented are the mean and standard deviation, the median, and 1. and 3. quantile. To test for the significance of changes between before and after therapy in the respective groups the Wilcoxon-Signed Rank Test was used. Statistically significant changes were bold-type.

**Supplementary Table 2:** Diversity parameters before and after therapy for the placebo and antibiotic group

|  | **Placebo**(n=27) | | | **Antibiotic**(n=27) | | |
| --- | --- | --- | --- | --- | --- | --- |
|  | **Baseline** | **2 months after therapy** | ***p*-value**  **before vs. after therapy** | **Baseline** | **2 months after therapy** | ***p*-value**  **before vs. after therapy** |
| **Richness** | 161.81 ± 53.27 | 169.22 ± 53.34 | *p*=0.768 | 169.93 ± 57.32 | 140.31 ± 62.72 | ***p*=0.002** |
|  | 164.74 (115.20 /  195.19) | 171.98 (130.72 / 205.64 |  | 179.56 (130.44  / 211.34) | 150.60 (85.32 /  196.86) |  |
| **Evenness** | 0.76 ± 0.04 | 0.79 ± 0.03 | ***p*=0.002** | 0.76 ± 0.05 | 0.75 ± 0.05 | *p*=0.202 |
|  | 0.76 (0.74 / 0.78) | 0.78 (0.76 / 0.81) |  | 0.77 (0.74 / 0.79) | 0.75 (0.73 / 0.78) |  |
| **Diversity** | 3.84 ± 0.39 | 4.00 ± 0.36 | *p*=0.090 | 3.86 ± 0.41 | 3.62 ± 0.57 | ***p*=0.016** |
|  | 3.88 (3.49 / 4.22) | 3.99 (3.70 / 4.26) |  | 3.86 (3.63 / 4.12) | 3.71 (3.27 / 4.03) |  |
| **Dissimilarity** | 0.70 ± 0.04 | 0.73 ± 0.05 | ***p*=0.034** | 0.71 ± 0.07 | 0.81 ± 0.08 | ***p*<0.001** |
|  | 0.69 (0.68 / 0.71) | 0.71 (0.70 / 0.75) |  | 0.69 (0.67 / 0.72) | 0.81(0.75 / 0.86) |  |

Richness, evenness, and diversity for the placebo and antibiotic group at baseline and 2 months after therapy. Presented are the mean and standard deviation, the median, and 1. and 3. quantile. To test for the significance of the changes between before and after therapy in the respective groups the Wilcox-Signed Rank Test was used. Statistically significant changes were bold-type.

| **Run number** | **run 1** | **run 2** |
| --- | --- | --- |
| **Q30 value [%]** | 76 | 82 |
| **Sensitivity [%]** | 100 | 100 |
| **False positives [n]** | 2 | 6 |
| **False positives [read%]** | 0.44 | 0.22 |

**Supplementary Table 3.:** Run quality control parameters

Illumina Q30 value, Sensitivity to find all 24 RSVs of the mock sample, number of false positives not occurring in the mock sample and percentages of false positive reads for the sequencing runs

**SUPPLEMENTARY FILES:**

**Supplementary File 1:** Mock composition with details of taxonomic classification down to subspecies, culture collection number, synonym, accession number, genome size, annotated 16S RNAs, 16S variants in V4 region, primer mismatches, and amplicon lengths per mock species (n=23).

### Supplementary File 2: Metadata containing sample-ID, anonymized patient identifier (patient-ID), treatment time point (before or after therapy), treatment group (antibiotic or placebo group) per sample (n = 54).

### Supplementary File 3: RSV abundance per sample with information about RSV-ID, read counts for each sample-ID, and sequence per aRSV (n = 1066).

### Supplementary File 4: Results of the negative binomial regression model (Deseq2) RSV-ID, log2FoldChange, standard error of log2FoldChange (lfcSE), adjusted p-values (padj) (n = 1066).

Adjusted p-values (according to Benjamini-Hochberg) are derived from a negative binomial regression model comparing aRSV abundances before and after treatment within each group. Taxonomic classifications are colored according to the Soccransky *et al*. complex affiliation. Unclassified = no taxonomic classification at this level possible; NA = not available, p_adj_ filtered out due to default independent filtering of DESeq2.
